# An effective colorimetric and ratiometric fluorescent probe based FRET with a large Stokes shift for bisulfite

**DOI:** 10.1038/srep25315

**Published:** 2016-05-03

**Authors:** Wen-Li Wu, Zhao-Yang Wang, Xi Dai, Jun-Ying Miao, Bao-Xiang Zhao

**Affiliations:** 1 Institute of Organic Chemistry, School of Chemistry and Chemical Engineering, Shandong University, Jinan 250100, P. R. China; 2 Institute of Developmental Biology, School of Life Science, Shandong University, Jinan 250100, P. R. China

## Abstract

Bisulfite plays crucial roles in diverse physiological processes. Therefore, the efficient detection of bisulfite is very important. In this study, we report a colorimetric and ratiometric fluorescent probe (CPT) with a large Stokes shift (162 nm) for bisulfite (HSO_3_^−^) based FRET mechanism. The probe can quantitatively detect HSO_3_^−^ with low detection limit (45 nM) and high specificity over other common anions and biothiols. A nucleophilic addition reaction was proposed for the sensing mechanism, which was confirmed by HRMS spectra. The test strips of the probe were made and used easily. Moreover, probe CPT was used to ratiometric fluorescent imaging of exogenous and endogenous HSO_3_^−^ in living cells.

Bisulfite (HSO_3_^−^), widely used as food preservative because of its antimicrobial, bacteriostasis and antioxidant property^1^, is found that its certain concentration level is responsible for respiratory diseases^2^,^3^. Therefore, more analytical methods should be developed for trace HSO_3_^−^. So far, many analytical methods including electrochemistry, chromatography, absorption and fluorescence spectroscopy have been developed to detect HSO_3_^−^ quantitatively^4^,^5^,^6^,^7^,^8^. Among these, fluorescence probes are widely applied because of their high selectivity, low detection limit and suitability for real-time monitoring.

Ratiometric fluorescent probes allowing the measurement of emission intensities at two different wavelengths could overcome the limitations of intensity-based probes and provide a self-calibration correction^9^,^10^,^11^,^12^,^13^. One well established method for developing ratiometric probes is based Förster Resonance Energy Transfer (FRET)-an excited-state energy interaction between two fluorophores, wherein the emission profile of one fluorophore (the donor) shows a significant overlap with the excitation profile of the other fluorophore (the acceptor). The ratio emission signal is modulated by the FRET process. On the other hand, a few probes with long emission wavelength based on 2-dicyanomethylene-3-cyano-4,5,5-trimethyl-2,5-dihydrofuran (TCF) have been reported because its excellent optical properties^14^,^15^,^16^,^17^,^18^,^19^,^20^,^21^,^22^.

Our group has introduced a new strategy to construct ratiometric fluorescent probes based on FRET^23^. The FRET process induced to prohibit the donor fluorescence and enhance the acceptor fluorescence. Upon reacting with the analytes, the FRET process will be interrupted to restore the donor fluorescence. As a continuation of previous work, we report a FRET-based fluorescent probe composed of coumarin-piperazine-TCF conjugate platform for the colorimetric and ratiometric detection of HSO_3_^−^.

## Results and Discussion

### UV-vis absorption and fluorescence property of probe CPT

**Figure 1:**
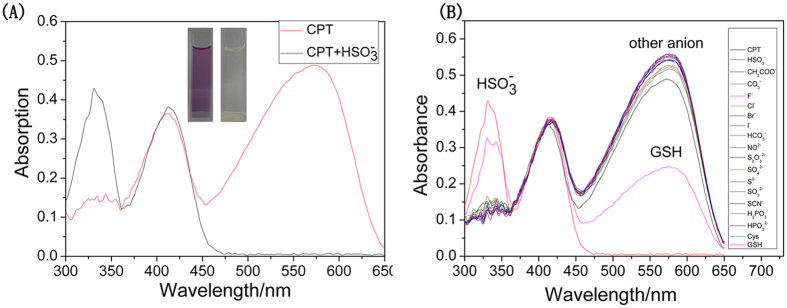
UV-vis absorption property of probe CPT. (**A**) UV-vis absorption spectra of probe **CPT** (10 μM) in the absence and presence of 10 equiv. of HSO_3_^−^ (Inset: the color change of CPT with or without HSO_3_^−^); (**B**) probe **CPT** with various analytes (100 equiv.) in EtOH-H_2_O solution (6:4 v/v, 10 mM PBS, pH 8.0).

We investigated firstly the effect of water content on fluorescence spectra of the probe. The reactivity of bisulfite toward the probe was sensitive over wide water content. Considering its’ best fluorescence ratio response as well as better peak shapes, we chose the solvent ratio (EtOH/H_2_O = 6:4) as the *vitro* test solvent system (Supplementary Fig. S1). The absorption maximum of free probe CPT centered at 570 nm and 410 nm, which were assigned to TCF and coumarin moiety, respectively (Fig. 1A). When various analytes (CH_3_CO_2_^−^, CO_3_^2−^, F^−^, Cl^−^, Br^−^, I^−^, HCO_3_^−^, NO_2_^−^, S_2_O_3_^2−^, NO_3_^−^, SO_4_^2−^, S^2−^, SO_3_^2−^, SCN^−^, H_2_PO_3_^−^, HPO_3_^2−^, Cys and GSH) were added into the solution of probe CPT, no significant absorption peaks change occurred for solutions with these analytes except for HSO_3_^−^ (Fig. 1B). In the case of biothiols (GSH, Cys), although their thiol groups are nucleophilic, they possess higher pK_a_ values (Cys 8.30, GSH 9.20). Besides, the electron-poor C = C group was often used as a reaction site to discriminate sulfite from other anion species through different activities in nucleophilic addition reaction. Upon addition of HSO_3_^−^, the maximum absorption peak at 330 nm appeared, and the maximum absorption peak at 570 nm disappeared, accompanying with the color change of solution from purple to colorless, suggesting that the conjugation system of the probe was interrupted due to the nucleophilic attack of HSO_3_^−^. So, probe CPT could serve as a “naked-eye” probe for HSO_3_^−^. Encouraged by the results, we applied probe CPT in test strips detection (Supplementary Fig. S2).

**Figure 2:**
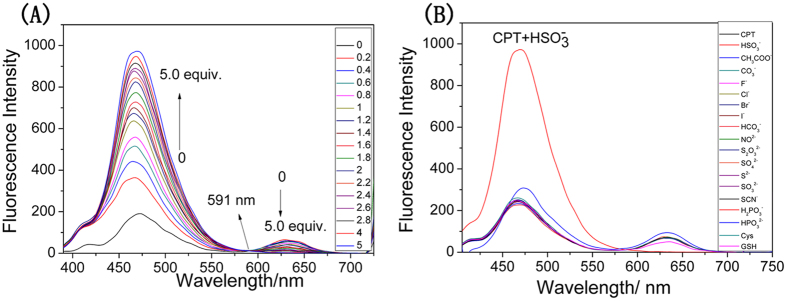
Fluorescence titration spectra of CPT. (**A**) Fluorescence titration spectra of **CPT** (2.5 μM) with incremental concentration of HSO_3_^−^ (0–5 equiv.). Data are mean SE (bars) (n = 3); (**B**) Fluorescence spectra of probe **CPT** in the presence of various analytes (100 equiv.) in EtOH-H_2_O solution (6:4 v/v, 10 mM PBS, pH 8.0).

To examine the ability of probe CPT to sense HSO_3_^−^, the fluorescence titration was conducted. The probe (2.5 μM) alone displayed two obvious fluorescence bands at 470 nm and 632 nm (fluorescence quantum yield *Φ* = 0.39, rhodamine B as standard), which attributed to coumarin and TCF unit, respectively (Supplementary Fig. S3). Upon addition of HSO_3_^−^ incrementally from 0 to 5 equiv., fluorescence emission band at 470 nm (*Φ* = 0.072, quinine sulfate as standard) increased gradually while the fluorescence at 632 nm decreased (Fig. 2A), which implies the acceptor moiety was destroyed by the reaction of the probe with HSO_3_^−^. The maximum emission peak underwent a 162 nm blue shift (from 632 nm to 470 nm) with an isoemission point at 589 nm, accompanying the fluorescence change from red to blue. These distinct responses confirmed that the FRET process was regulated by the reaction of the probe with HSO_3_^−^. The ratios of emission intensity at 470 and 632 nm (I_470_/I_632_) exhibited a drastic change from 2.841 to 546.986, a 192-fold enhancement with increasing concentrations of HSO_3_^−^. On the other hand, the probe showed no fluorescence intensity change toward other analytes (Fig. 2B). Further, we explored the interference of these analytes. The results demonstrated that probe CPT had high selectivity toward HSO_3_^−^ and had almost no interference from other analytes (Supplementary Fig. S4).

Moreover, we also conducted the absorption titration experiments (Supplementary Fig. S5). Upon addition of HSO_3_^−^ to the solution of probe CPT, new absorption bands at 330 nm enhanced consistently with the increasing concentrations (0–3 equiv.). Meanwhile, absorption bands at 410 nm were almost no change, however, absorption bands at 570 nm decreased. The results implied the reaction of TCF moiety with HSO_3_^−^, which was consistent with that from fluorescence titration. Ratiometric signaling of the fluorescence output of the probe (2.5 μM) at two different wavelengths indicated that the ratios reached a plateau when 15 μM of HSO_3_^−^ was added (Supplementary Fig. S6). In addition, there was a linear correlation between the fluorescence intensity ratios and the concentrations of HSO_3_^−^ from 0 to 7 μM, and the limit of detection (LOD) was calculated to be 45 nM, which is superior to most reported probes (Supplementary Table S1).

As an important factor for evaluating the probe in practical sensing, response time toward HSO_3_^−^ was tested (Supplementary Fig. S7). Clearly, the ratios of emission intensity reached maximum at around 1 h, indicating the reaction accomplished. In the study, we utilized 10 equivalents (relatively lower) of HSO_3_^−^, causing relatively rapid response (Supplementary Table S1). To obtain information concerning the pH effects, lg(I_470_/I_632_) changes of probe CPT (2.5 μM) was investigated at different pH (Supplementary Fig. S8). The results indicated that the probe was stable and can function well over a wide range of relatively alkaline pH (7.0–10.0). As expected, there was hardly any response to HSO_3_^−^ in acidic condition, which could be demonstrated by the facts that HSO_3_^−^ cannot exist under stronger acid conditions.

### Recognition mechanism

**Figure 3:**
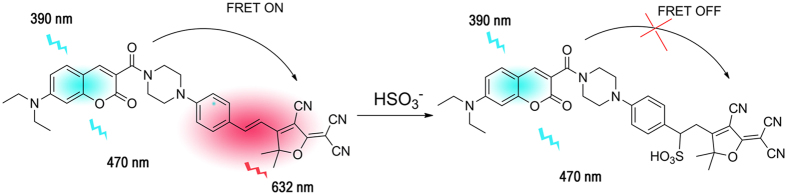
Proposed sensing mechanism of probe CPT for HSO_3_^−^.

The supposed ratiometric fluorescence mechanism is shown in Fig. 3. For the probe alone, FRET between the coumarin and the TCF moiety resulted in red emission from the TCF acceptor with the excitation of coumarin moiety. Upon the addition of HSO_3_^−^, the energy transfer from coumarin donor to TCF acceptor was interrupted because of the breakage of C = C bond by the addition reaction with HSO_3_^−^. The reaction product of the probe with HSO_3_^−^ displayed coumarin emission.

To confirm the FRET mechanism, we synthesized the donor and the acceptor (Supplementary). The addition of HSO_3_^−^ to the donor caused no fluorescence change at 470 nm, while the addition of HSO_3_^−^ to the acceptor quenched the fluorescence at 640 nm (Supplementary Fig. S9). These results confirmed that the conjugated structure of the acceptor was interrupted. The MS spectra of the reaction product between the acceptor and HSO_3_^−^ also supported the deduction (calcd for C_22_H_24_N_5_O_4_S: 454.52, found 454.50) (Supplementary Fig. S10). Moreover, the overlap between the absorption spectra of the acceptor and the emission spectra of the donor showed that the FRET process occurred though the intramolecular energy transfer efficiency is not so effectively (44.5%) (Supplementary Fig. S11). Pleasantly, a large emission shift (162 nm) with two-well resolved emission bands (632/470 nm) before and after interaction with HSO_3_^−^ generated, which could ensure accuracy in determining their intensities and ratios^24^. Finally, to further clarify the proposed mechanism, HRMS of the reaction product from probe CPT and HSO_3_^−^ was carried out. The results confirmed that the treatment of probe CPT with HSO_3_^−^ could afford nucleophilic addition product, accompanying a new peak appeared at 721.2068 (Supplementary Fig. S12).

### Cell imaging of probe CPT

CPT was of low cytotoxicity (Supplementary Fig. S13) and had excellent photostability (Supplementary Fig. S14). Encouraged by the aforementioned results, we further investigated the applicability of CPT for exogenous and endogenous HSO_3_^−^ detection in Hela cells. Staining Hela cells with CPT gave strong fluorescence in red channel and weak fluorescence in the blue channel. Further incubated with NaHSO_3_ the cells displayed a distinct fluorescence increase in blue channel, accompanied by the dramatic fluorescence drop in red channel (Supplementary Fig. S15). These results indicated that CPT can provide ratiometric detection for HSO_3_^−^ in cells.

**Figure 4:**
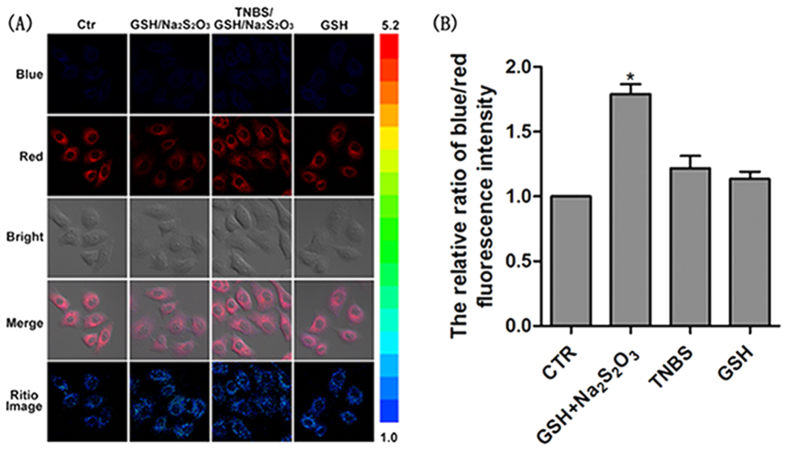
(**A**) The first row (vertically): HepG2 cells were incubated with CPT (5 μM) for 40 min; The second row: HepG2 cells were incubated with 500 μM GSH and 250 μM Na_2_S_2_O_3_ for 1 h, and then with CPT (5 μM) for 40 min; The third row: HepG2 cells were incubated with 10 mM TNBS (2,4,6-trinitrobenzenesulphonate, known as a TST inhibitor) for 0.5 h, then with 500 μM GSH and 250 μM Na_2_S_2_O_3_ for another 1 h, followed by CPT (5 μM) for 40 min; The fourth row: HepG2 cells were incubated with 500 μM GSH for 1 h, then with CPT (5 μM) for another 40 min. (**B**) From left to right: the relative ratio of blue/red fluorescence intensity of row 1, 2, 3 and 4 in (A). The ratio images were all obtained as F_blue_/F_red_. Images were acquired from 405–555 nm for blue fluorescence, and from 560–700 nm for red fluorescence. λex = 405 nm.

Further, we investigated the response of CPT toward endogenous HSO_3_^−^ in cells. Endogenous HSO_3_^−^ in cells can be produced from thiosulfate via thiosulfate sulphurtransferase (TST), which is abundant in mammalian liver cells^25^. Therefore, HepG2 cells (human liver cancer cells) and L-02 cells (human normal liver cells) were chosen to investigate the capability of this probe for the detection of endogenous bisulfite. A clear fluorescence change was observed only when HepG2 cells were incubated with CPT (5 μM) for 1 h followed by incubating with 500 μM GSH and 250 μM Na_2_S_2_O_3_ for 0.5 h (Fig. 4). These results demonstrated that the probe was capable of detecting endogenous bisulfite in HepG2 cells. However, no significant fluorescence change was observed in L-02 cells incubated with probe CPT (5 μM) and GSH/Na_2_S_2_O_3_ (Supplementary S16). The comparison between these results demonstrated that CPT could potentially be applied to differentiate liver cancer cells and normal liver cells.

## Conclusions

In summary, we have developed a new colorimetric and ratiometric fluorescent probe based on FRET between coumarin and TCF fluorophor with a large Stokes shift (162 nm) for detecting HSO_3_^−^. The probe exhibits a clear HSO_3_^−^ induced change in the intensity ratio of the two emission bands of coumarin and TCF, with high selectivity and sensitivity. The probe has a detection limit as low as 45 nM, which is superior to most reported probes. Moreover, the probe was used to ratiometric fluorescent imaging of endogenous HSO_3_^−^ in living cells.

## Methods

### Apparatus and chemicals

Melting points were measured on an XD-4 digital micro-melting point apparatus. ^1^H NMR and ^13^C NMR spectra were recorded on a Bruker Avance 300 MHz spectrometer. HRMS spectra were obtained on a Q-TOF6510 spectrograph (Agilent). UV-vis spectra were measured by using a Hitachi U-4100 spectrophotometer. Twice-distilled water was used throughout all experiments. All the pH measurements were made with a PHS-3C pH meter. Thin-layer chromatography (TLC) was conducted on silica gel 60 F_254_ plates (Merck KGaA) and column chromatography was conducted over silica gel (mesh 200–300). All of fluorescence spectra were obtained by the excitation at 390 nm, slit 8/9 nm. All the samples were investigated in EtOH-H_2_O solution (6:4 v/v, 10 mM PBS, pH 8.0). Quartz cuvettes with a 1 cm path length and 3-mL volume were involved in fluorescence and UV -vis spectra measurements. All reagents were purchased from J&K, Aladdin and Sinopharm Chemical Reagent Co. and used without further purification.

### Cell culture and cell imaging of the probe in HepG2 cells and L-02 cells

Hela cells were cultured in a 6-well plate in Dulbecco’s modified Eagle’s medium (DMEM) supplemented with 10% fetal bovine serum in an atmosphere of 5% CO_2_ and 95% air at 37 °C.

HepG2 cells or L-02 cells were cultured in a 6-well plate in DMEM supplemented with 10% fetal bovine serum in an atmosphere of 5% CO_2_ and 95% air at 37 °C. The probe CPT was dissolved in DMSO to get the stock solution (10 mM) and diluted to 5 μM before use. HepG2 cells or L-02 cells were incubated with a mixture of 500 μM GSH and 250 μM Na_2_S_2_O_3_ for 1 h, and then treated with 5 μM CPT for 40 min. For control experiments, CPT loaded HepG2 cells were pretreated with 10 mM TNBS for 0.5 h, and then treated with a mixture of 500 μM GSH and 250 μM Na_2_S_2_O_3_ for another 1 h. Simultaneously, CPT loaded HepG2 cells were treated with 500 μM GSH only for 40 min. Subsequently, excited at 405 nm, the cells were imaged under a confocal microscope (LSM 700) and the images were collected at emission channels of 405–555 nm (blue channel) and 560–700 nm (red channel), respectively.

### Statistical analysis

Data are presented as mean ± SEM and analysis involved the use of GraphPad Prism 5. Images were processed by Adobe Photoshop CS5 (Adobe, San Jose, USA). P < 0.05 was considered statistically significant.

## Additional Information

**How to cite this article**: Wu, W.-L. *et al.* An effective colorimetric and ratiometric fluorescent probe based FRET with a large Stokes shift for bisulfite. *Sci. Rep.* **6**, 25315; doi: 10.1038/srep25315 (2016).

## Supplementary Material

Supplementary Information
