# Supplementary material for: An effective colorimetric and ratiometric fluorescent probe based FRET with a large Stokes shift for bisulfite
Source: Sci Rep. 2016 May 3;6:25315. doi: 10.1038/srep25315 (PMC4853739; doi:10.1038/srep25315)
Supplement: Supplementary Information [file srep25315-s1.pdf]

Electronic Supplementary Information

An effective colorimetric and ratiometric fluorescent probe based FRET with a large Stokes shift for bisulfite

Wen-Li Wu[1, ‡], Zhao-Yang Wang[2, ‡], Xi Dai[1], Jun-Ying Miao[2,*], Bao-Xiang Zhao[1,*]

[1]Institute of Organic Chemistry, School of Chemistry and Chemical Engineering, Shandong University, Jinan 250100, P.R. China.

[2] Institute of Developmental Biology, School of Life Science, Shandong University, Jinan 250100, P.R. China.

[*]Correspondence to: Prof. BaoXiang Zhao and Prof. JunYing Miao

Fax: + 86 531 88564464; Tel.: + 86 531 88366425.

E-mail address: bxzhao@sdu.edu.cn, miaojy@sdu.edu.cn,

[‡] Equal contribution

Methods and Synthesis

Fluorescence quantum yield

Calculation of energy transfer efficiency

Calculation of the detection limit (LOD)

Synthesis of the probe, donor, acceptor

Preparation for UV - vis and fluorescence spectral measurements

Cytotoxicity Assay

Preparation of probe CPT test strips

Test strips application

Comparison of ratiometric fluorescent probes for HSO_3^-/SO_3^{2-} (Table S1)

Methods and Synthesis

Fluorescence quantum yield

Fluorescence quantum yield was determined by the relative comparison with quinine sulfate (Φ_s = 0.56 in 0.1 N H_2SO_4 aqueous solution) and rhodamine B (Φ_s = 0. 69 in ethyl alcohol solution) as standard, and it was calculated by equation following.

$$\Phi = \Phi_s(IA_s/I_sA)(\eta^2/\eta_s^2) \qquad (1)$$

in which, *A* is the absorbance, *I* is the integrated fluorescence intensity, and *η* is the refractive index of the solvent.

Calculation of energy transfer efficiency

Energy transfer efficiency (E) was calculated using the following equation:

$$E = 1 - F_{DA} / F_D \qquad (2)$$

Where, F_{DA} and F_D denote the donor fluorescence intensity with and without an acceptor, respectively.

Calculation of the detection limit (LOD)

$$LOD = 3\sigma/k \qquad (3)$$

Where, σ is the standard deviation of the blank solution and k is the slope of the linear calibration plot between the fluorescence emission intensity and the concentration of HSO_3^-.

Synthesis of 2-(3-cyano-4-(4-(4-(7-(diethylamino)-2-oxo-2H-chromene-3-carbonyl)piperazin-1-yl)styryl)-5,5-dimethylfuran-2(5H)-ylidene)malononitrile (probe CPT)

2-(3-Cyano-4,5,5-trimethylfuran-2(5H)-ylidene)malononitrile (TCF) was synthesized according to literature methods from 3-hydroxy-3-methylbutan-2-one and malononitrile[1]. 4-(4-(7-(Diethylamino)-2-oxo-2H-chromene-3-carbonyl)piperazin-1-yl)benzaldehyde (200 mg, 0.462 mmol), TCF (110.3 mg, 0.554 mmol) were dissolved in absolute ethanol (20 mL) and refluxed for 1 h. After cooling to room temperature, the orange solid was filtered. The crude product was purified by column chromatography on silica gel using dichloromethane/methanol (10/1, v/v), to afford probe **CPT** (238.5 mg) in 84.3% yield. Red powder, m.p.: 232 – 234 ℃; ^{1}H NMR (300 MHz, DMSO-d_6): 8.03 (s, 1H, ArH), 7.91 (d, *J* = 15.9 Hz, 1H, ArH), 7.81 (d, *J* = 15.9 Hz, 2H, ArH), 7.51 (d, *J* = 9.0 Hz, 1H,

ArH), 7.05 (d, J = 9.3 Hz, 2H, ArH), 6.96 (d, J = 15.9 Hz, 1H, -CH=*CH*-), 6.76 (dd, J = 9.0 and 2.1 Hz, 1H, -*CH*=CH-), 6.57 (d, J = 2.1 Hz, 1H, ArH), 3.72-3.32 (m, 12H, ArH), 1.76 (s, 6H, -CH_3), 1.13 (t, J = 7.2 Hz, 6H, -CH_3); ^{13}C NMR (75 MHz, DMSO-d_6): 177.23, 175.55, 164.17 (2C), 158.36, 156.59, 153.29, 151.24 (2C), 148.60, 144.10, 132.34, 130.08, 123.64, 115.53, 113.89, 113.14, 112.31, 111.57, 109.90, 109.35, 107.04, 98.47, 96.21, 93.97, 55.91 (2C), 51.85 (2C), 44.08 (2C), 25.39, 18.45 (2C), 12.20 (2C). HRMS (m/z): $[M]^+$ calcd for $C_{36}H_{35}N_6O_4$, 615.2714, found 615.2673 (Fig. S17-S19).

Synthesis of 2-(3-cyano-5,5-dimethyl-4-(4-(piperazin-1-yl)styryl)furan-2(5H)-ylidene)malononitrile (acceptor)

The synthesis method is the same as the probe CPT. MS: m/z $[M]^+$ calcd for $C_{36}H_{35}N_6O_4$, 372.18, found 372.17 (Fig. S20).

Synthesis of 7-(diethylamino)-*N,N*-diethyl-2-oxo-2H-chromene-3-carboxamide (donor)

The synthesis method is according to the literature[2]. HRMS: m/z $[M]^+$ calcd for $C_{18}H_{25}N_2O_3$ 317.1865, found 317.1864 (Fig. S21- S23).

Preparation for UV -vis and fluorescence spectral measurements

Phosphate buffered saline (PBS, 10 mM) was used throughout the absorption and fluorescence determination. Probe CPT was dissolved in ethanol (EtOH) to get the stock solution (1×10^{-3} M). Twice-distilled water was used to prepare stock solution (1×10^{-2} M) of NaF, NaCl, NaBr, KI, $NaHCO_3$, KNO_2, Na_2SO_4, KSCN, $Na_2S_2O_3$, Na_2S, Na_2SO_3, $NaHSO_3$, Na_2CO_3, CH_3COONa, NaH_2PO_3, Na_2HPO_3, cysteine, and glutathione. Stock solution of $NaHSO_3$ and Na_2SO_3 was freshly prepared each time before use. Test solution was prepared by placing 25 μL of the stock solution and an appropriate aliquot of each testing species solution into a 10 - mL volumetric flask, and the solution was diluted to 10 mL with PBS buffer (10 mM, pH 8.0) containing 60% EtOH (v/v).

Cytotoxicity Assay

HeLa Cells were cultured in Dulbecco's modified Eagle's medium(DMEM) supplemented with 10%

FBS in an atmosphere of 5% CO_2 and 95% air at 37℃. The cells were placed in a 96-well plate, followed by addition of probe CPT with final concentrations of 1, 5, 10 μM, respectively. The cells were then incubated for 3 h, followed by SRB assays.

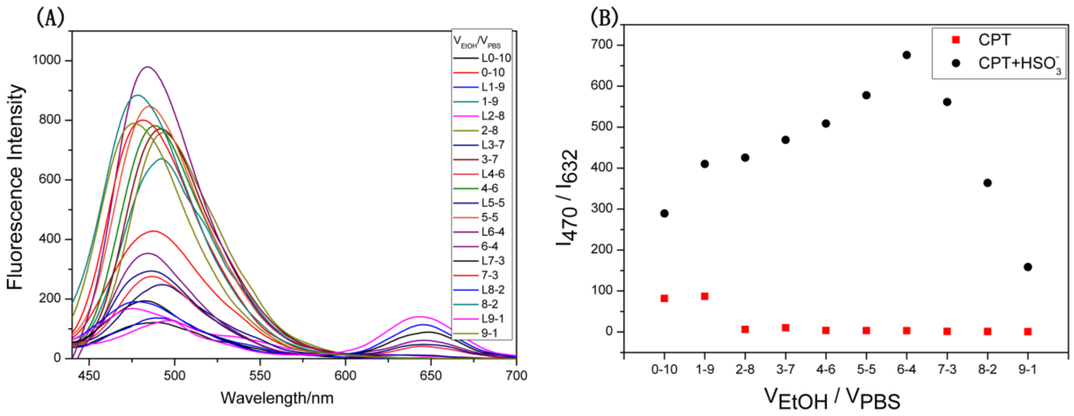

Figure S1: Fluorescence spectra of probe **CPT** (2.5 μM) with or without HSO_3^- (10 equiv.) in different water content (V_{EtOH}/V_{PBS}) (10 mM PBS, pH 8.0). (Ex = 390 nm, slit: 8/9 nm).

Preparation of CPT Test Strips

Probe **CPT** was dissolved in EtOH to afford the test solution (0.1 mg/mL, 10 mL). Filter paper was soaked in the test solution for 30 seconds, and then dried in a vacuum drying oven. 4 test strips were soaked in the 4 bisulfite solution (0, 10^{-4}, 10^{-3}, 10^{-2} M) for 2 min, respectively, then dried. The photographs were taken in visible light after 5 min.

Test strips application

Encouraged by the results, we made bisulfite test strips to detect different concentrations of bisulfite in water. With the concentration of HSO_3^- increasing (from 0, 10^{-4}, 10^{-3}, 10^{-2} M), the color of the test strips gradually faded from red in visual light (Fig. S2), which was consistent with that in solution. The results showed that probe CPT could sensitively and simply detect bisulfite in practical water sample with the naked eye.

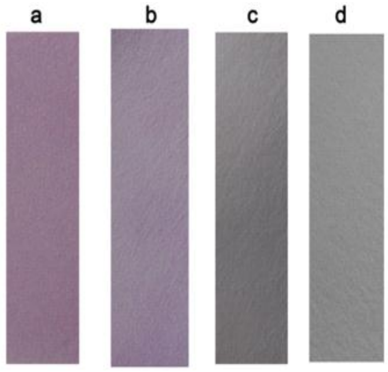

Figure S2: Test strips application of CPT. Visual changes of **CPT** (0.1 mg/mL)-coated test strips after soaked in different concentrations of HSO_3^- (a: 0, b: 1×10^{-4} M, c: 1×10^{-3} M, d: 1×10^{-2} M) in aqueous solution.

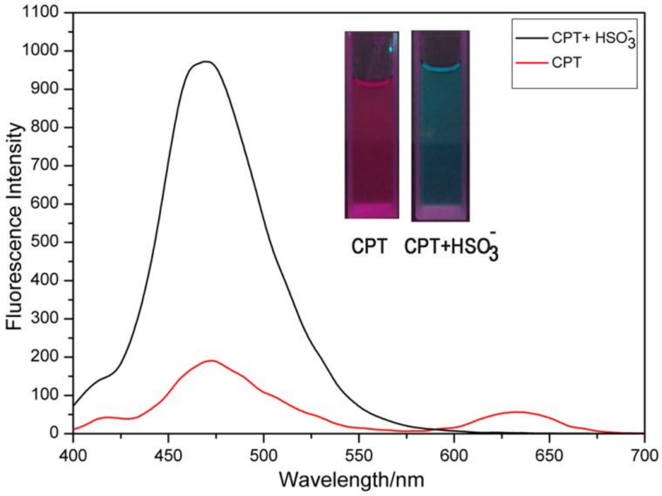

Figure S3 Fluorescence spectra of probe **CPT** (2.5 μM) in the absence and presence of 10 equiv. of HSO_3^- in EtOH-H_2O solution (6:4 v/v, 10 mM PBS, pH 8.0); Inset: the fluorescence change of CPT with or without HSO_3^- under 365 nm UV lamp of CPT in EtOH-H_2O solution (6:4 v/v, 10 mM PBS, pH 8.0).

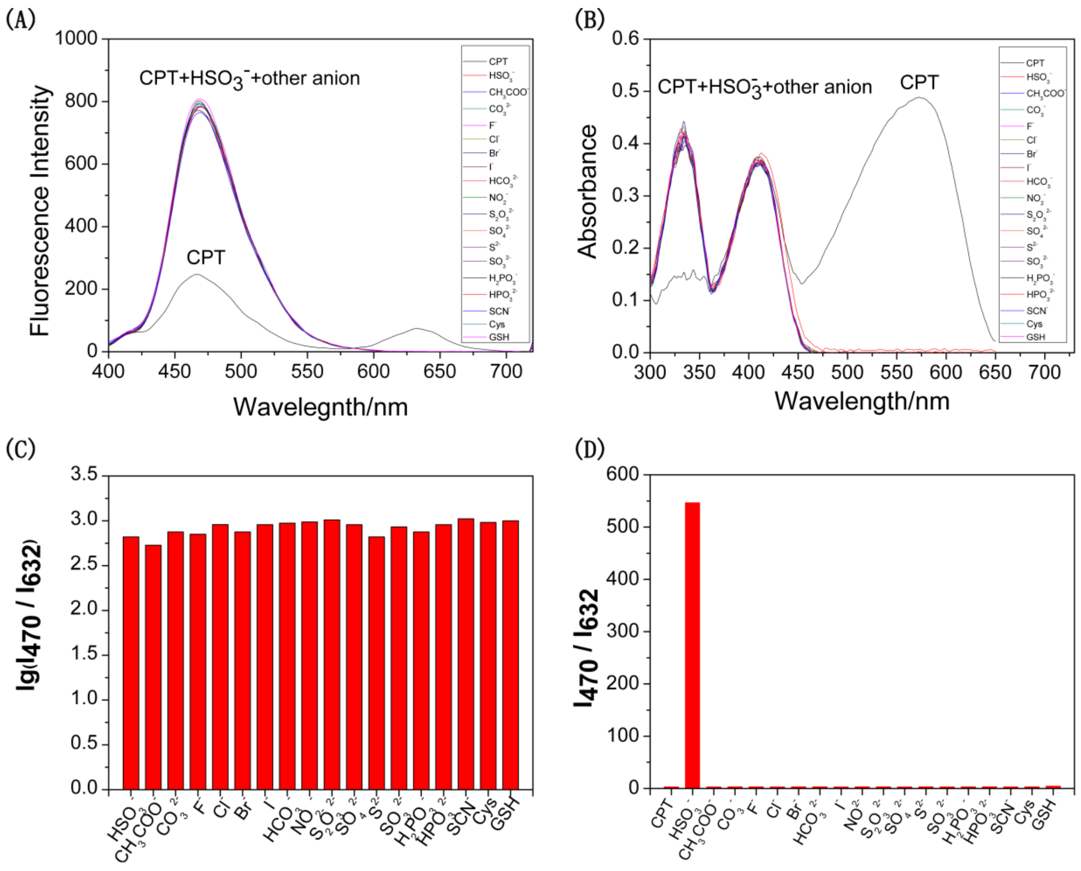

Figure S4 The selectivity of CPT towards various analytes. (A) Fluorescence spectra of CPT (2.5 μM) and (B) absorption spectra of CPT (10 μM) with HSO_3^- (10 equiv.) and various analytes (100 equiv.); (C) Ratiometric response of CPT with HSO_3^- (10 equiv.) in the presence of various analytes; (D) Ratiometric response of CPT with various analytes (100 equiv.) in EtOH-H_2O solution (6:4 v/v, 10 mM PBS, pH 8.0). (Ex = 390 nm, slit: 8/9 nm)

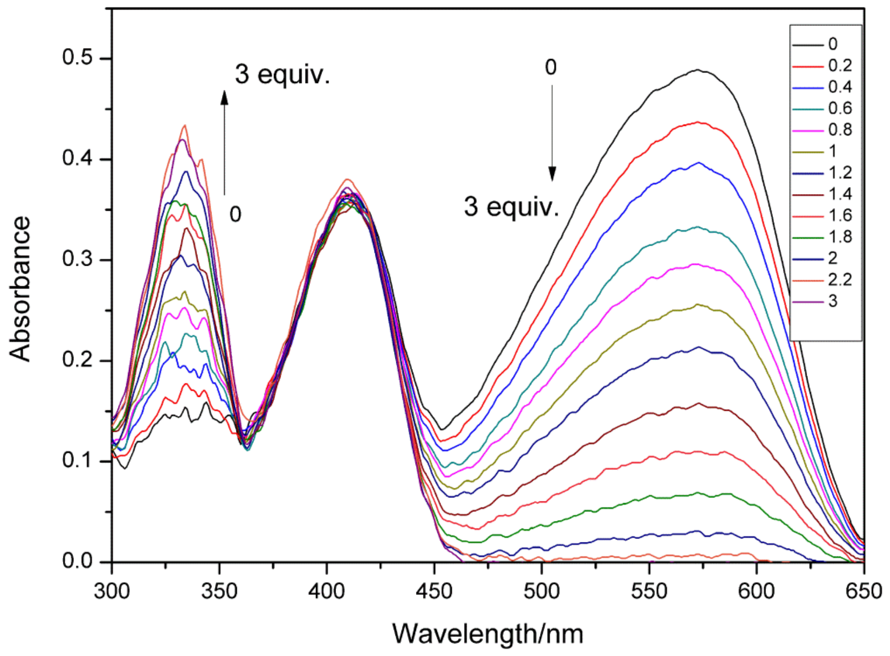

Figure S5 The absorption titration spectra of CPT (10 μM) upon the incremental addition of HSO_3^- (0-3 equiv.).

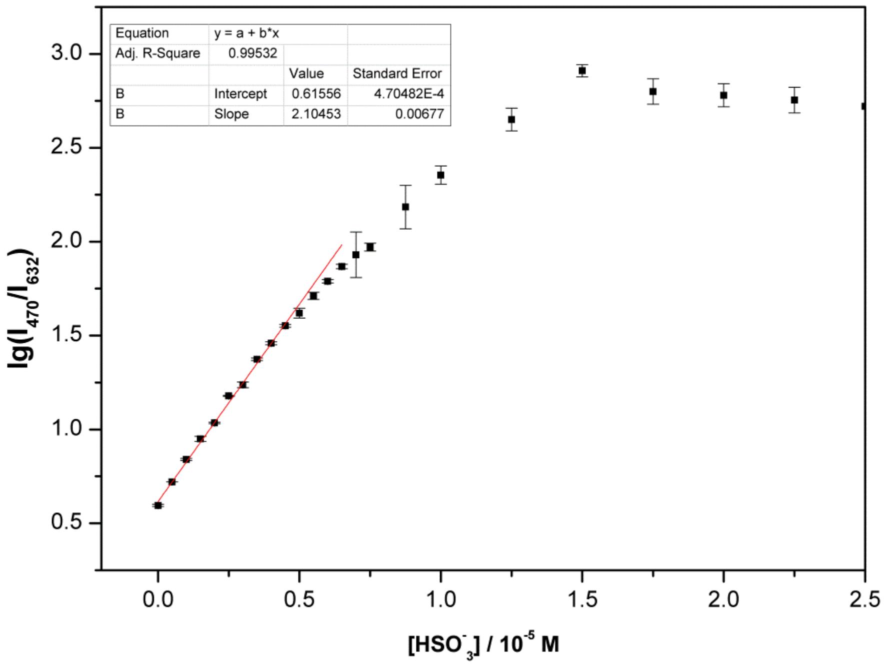

Figure S6 The plot of ratiometric responses (I_{470}/I_{632}) as a function of concentrations of HSO_3^-. Data are mean ±SE (bars) (n = 3). CPT (2.5 μM)

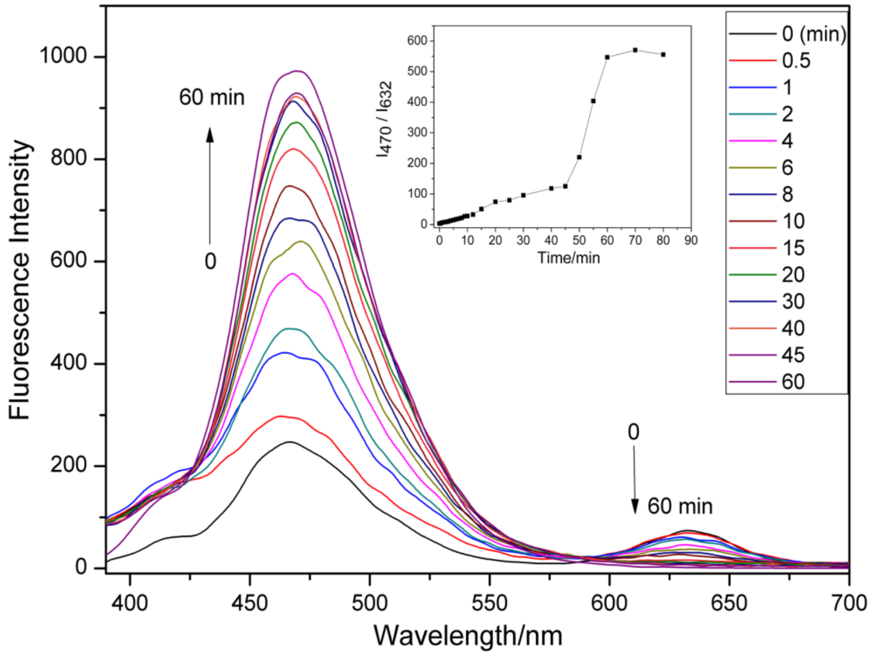

Figure S7 The time-dependence of CPT towards to HSO_3^-. CPT (2.5 μM), HSO_3^- (3 equiv.). Inset: The plot of ratiometric responses (I_{470}/I_{632}) as a function of time in EtOH-H_2O solution (6:4 v/v, 10 mM PBS, pH 8.0). (Ex = 390 nm, slit: 8/9 nm)

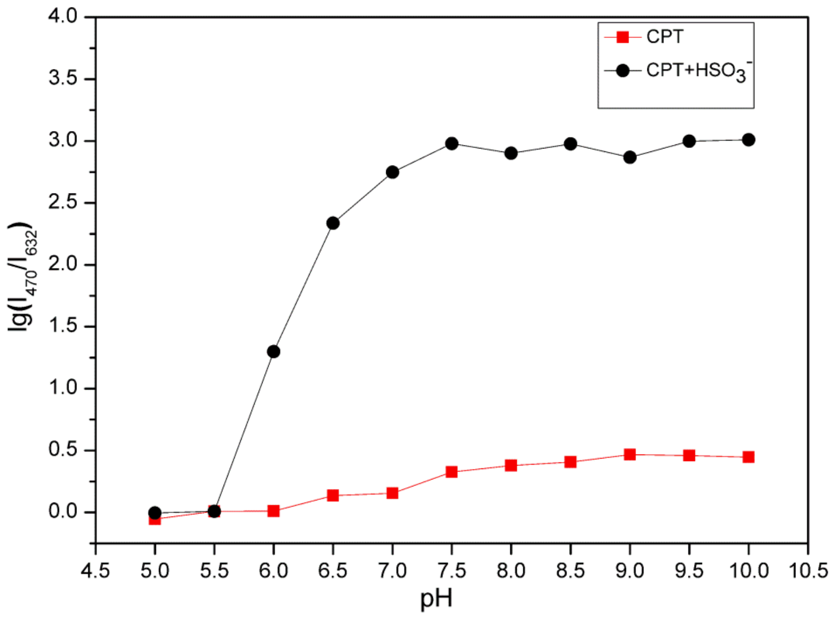

Figure S8 The pH-dependence of CPT towards to HSO_3^-. Fluorescence ratiometric response (I_{470}/I_{632}) of **CPT** (2.5 μM) with or without HSO_3^- (10 equiv.) in EtOH-H_2O solution (6:4 v/v, 10 mM PBS). (Ex = 390 nm, slit: 8/9 nm).

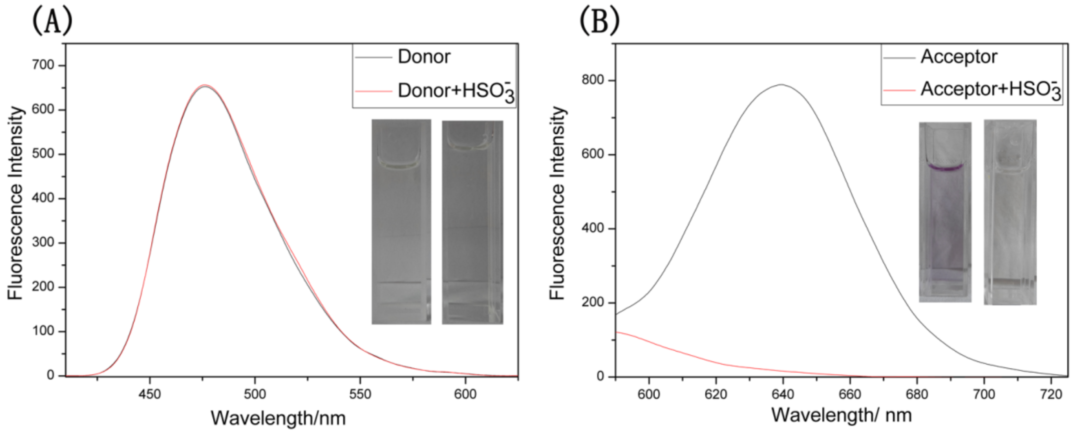

Figure S9: Emission spectral of the donor and the acceptor with HSO_3^-. (A) The emission spectral of the donor (10.0 μM) with or without HSO_3^- (10 equiv.) in EtOH-H_2O solution (6:4 v/v, 10 mM PBS, pH 8.0). (Ex = 390 nm, slit: 10/2.5 nm); (B) The emission spectral of the acceptor (10.0 μM) with or without HSO_3^- (10 equiv.) in EtOH-H_2O solution (6:4 v/v, 10 mM PBS, pH 8.0). (Ex = 550 nm, slit: 3/12 nm)

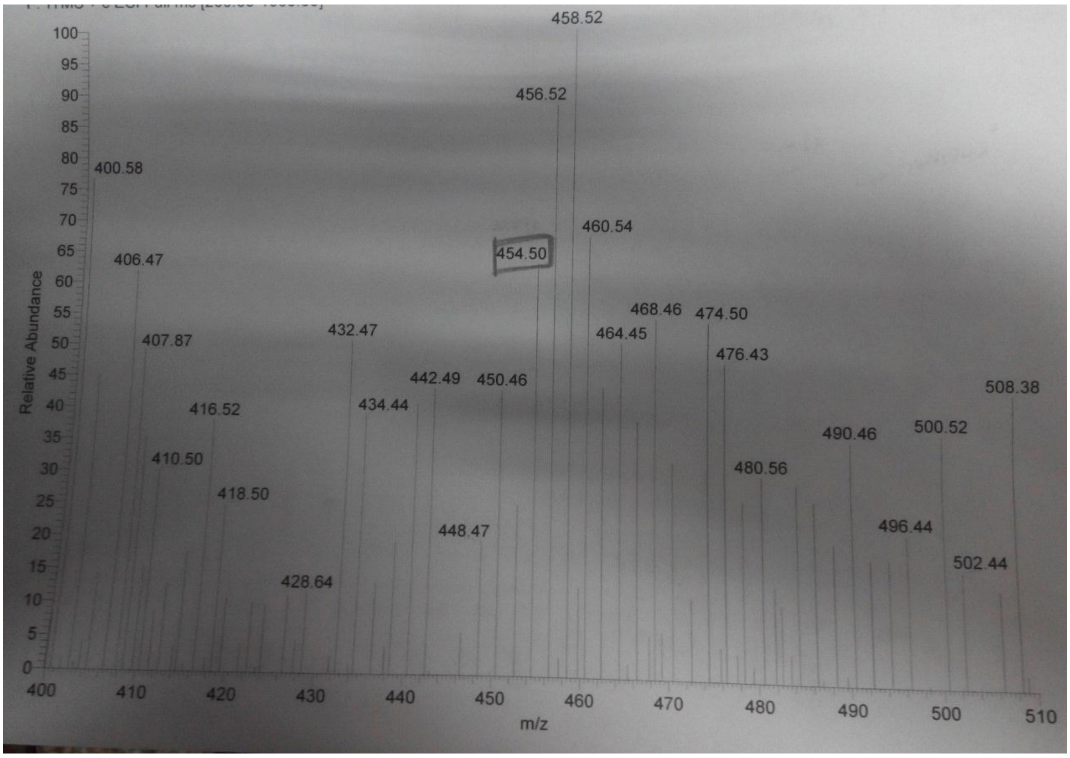

Figure S10 Mass spectrum of the conjugate addition product of the acceptor with HSO_3^-

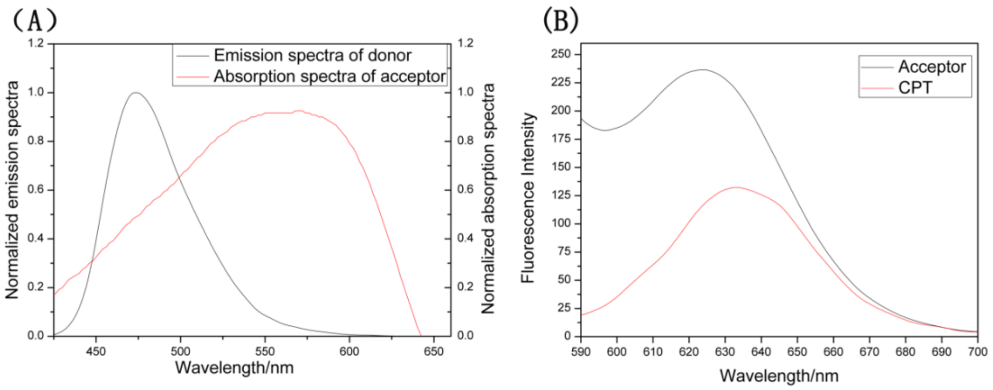

Figure S11 The energy transfer efficiency of FRET. (A) The normalized spectral overlaps between the energy donor emission and the acceptor absorption; (B) the emission spectral of probe CPT (2.5 μM) and the acceptor (2.5 μM) in EtOH-H_2O solution (6:4 v/v, 10 mM PBS, pH 8.0). (Ex = 390 nm, slit: 8/9 nm).

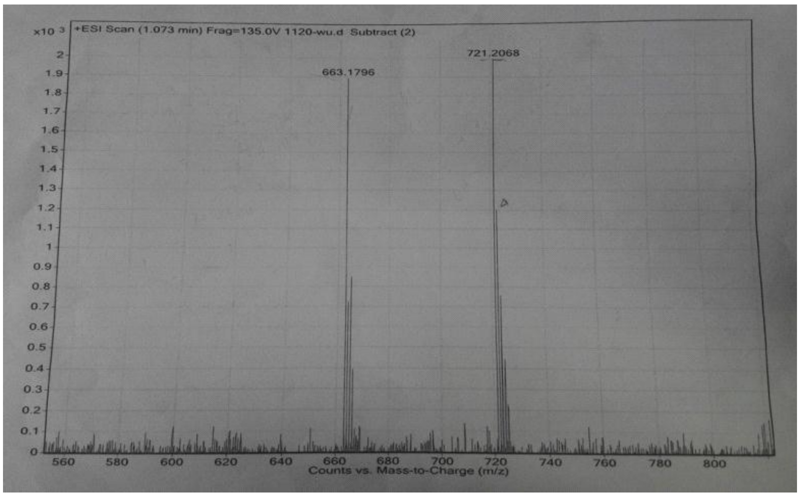

Figure S12 High resolution mass spectrum of the conjugate addition product of **CPT** with HSO_3^-

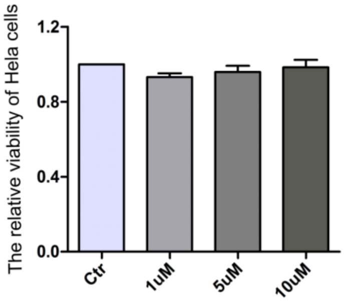

Figure S13 Viability of Hela cells incubated with probe **CPT** (0, 1, 5, 10 μM) for 3 h. Data are mean SE (bars) (n = 3).

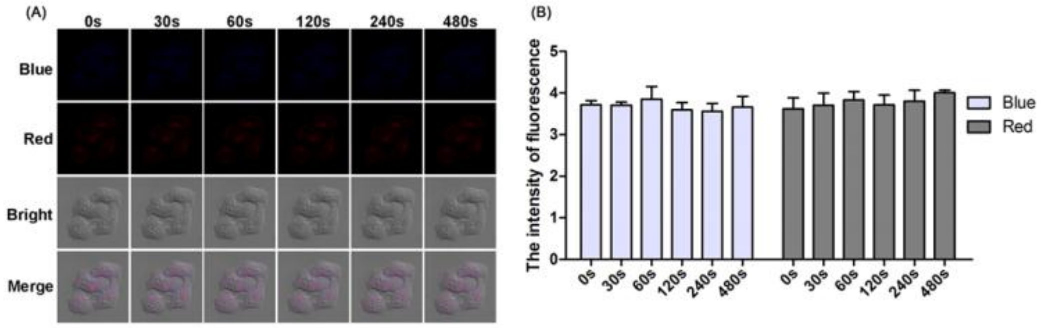

Figure S14 Photostability of probe CPT. (A) Photostability of probe **CPT** (5 μM). Fluorescence images (0 - 480 s) were achieved by means of time-sequential scanning of the Hela cells. (B) Fluorescence intensity of blue channel (405-555 nm, left) and red channel (560-700 nm, right) of (a) from 0 to 480 s.

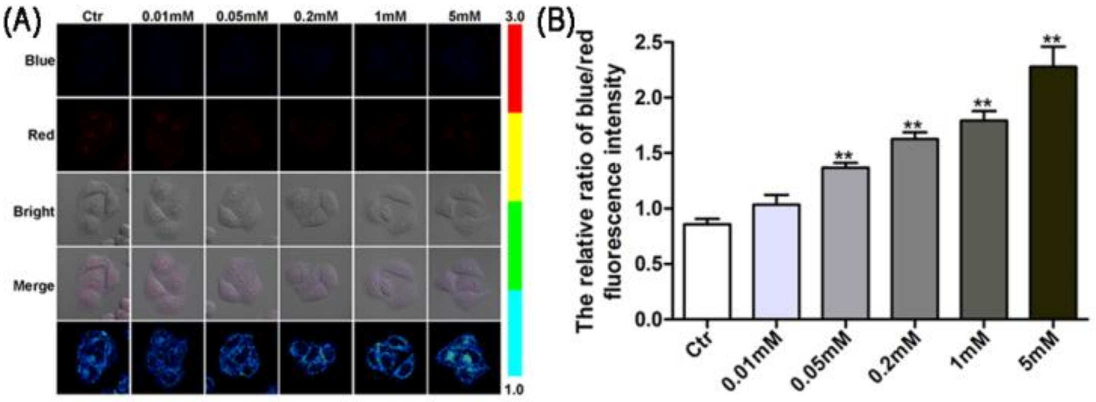

Figure S15 Confocal fluorescence imaging of HSO_3^- in Hela cells using probe CPT. Fluorescence imaging of Hela cells incubated with CPT (5 μM) for 1 h from confocal microscopy (LSM700) and then treated with $NaHSO_3$ (0.01, 0.05, 0.2, 1 or 5 mM) for 0.5 h. (A) fluorescence imaging from the blue channel, the red channel, bright field, overlay of blue, red and bright field, respectively; (B) The relative ratio of blue/red fluorescence intensity under different concentrations of $NaHSO_3$. Data are mean SE (bars) (n = 3, **, $p < 0.01$, λ_{ex} = 405 nm, blue channel 405-555 nm, red channel 560-700 nm).

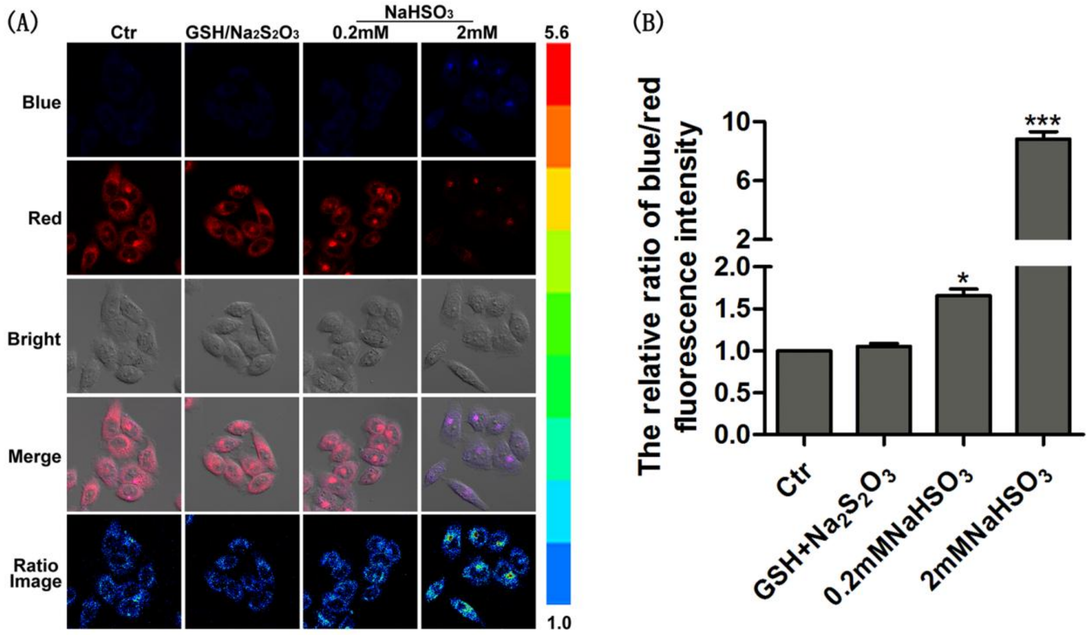

Figure S16 (A) The first row (vertically): L-02 cells were incubated with CPT (5 μM) for 40 min; The second row: L-02 cells were incubated with 500 μM GSH and 250 μM $Na_2S_2O_3$ 1 h, and then were incubated CPT (5 μM) for 40 min; The 3-4 row: L-02 cells were incubated with 0.2 and 2 mM $NaHSO_3$ for 1 h, and then with CPT (5 μM) for 40 min, respectively. (B) The relative ratio of blue/red fluorescence intensity of row 1-4 in (A). The ratio images were all obtained as F_{blue}/F_{red}. Images were acquired from 405-555 nm for blue fluorescence, and from 560-700 nm for red fluorescence. λex = 405 nm.

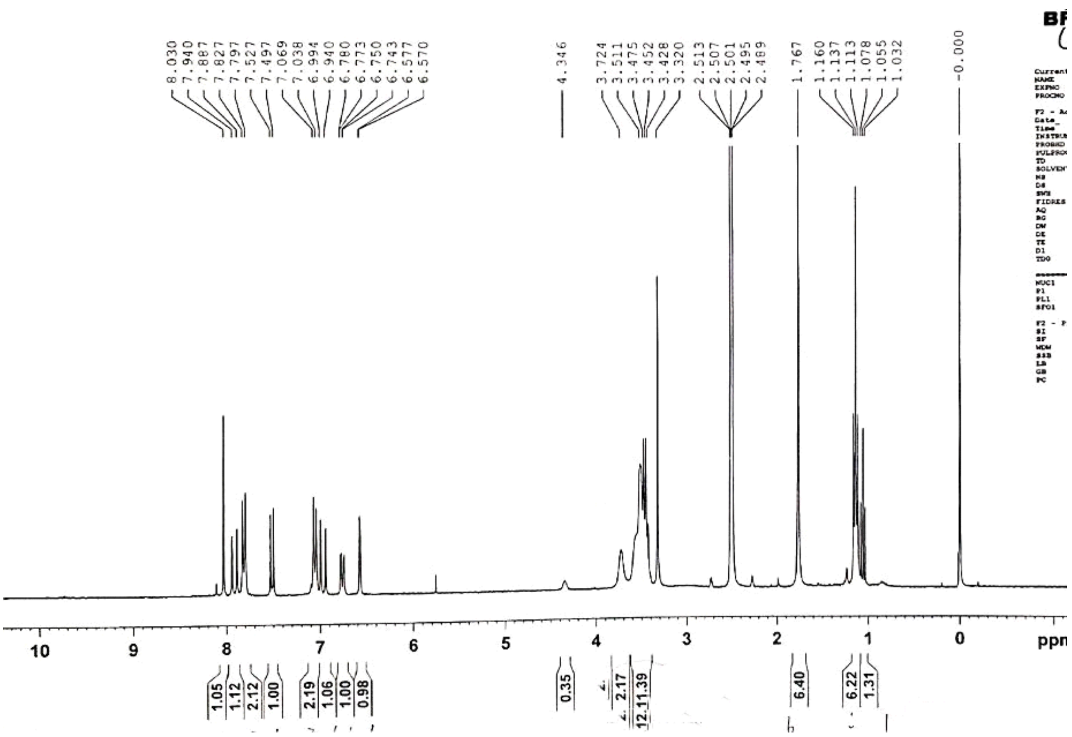

Figure S17 ^{1}H NMR spectrum of probe **CPT** (DMSO-d_6).

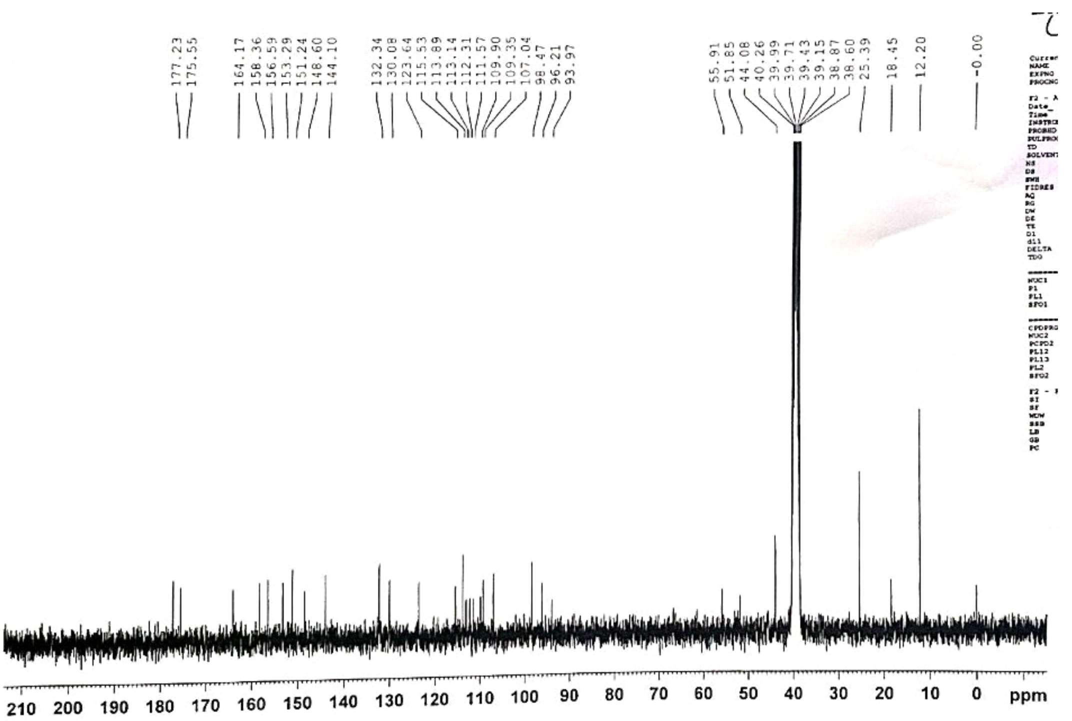

Figure S18 ^{13}C NMR spectrum of probe **CPT** (DMSO-d_6).

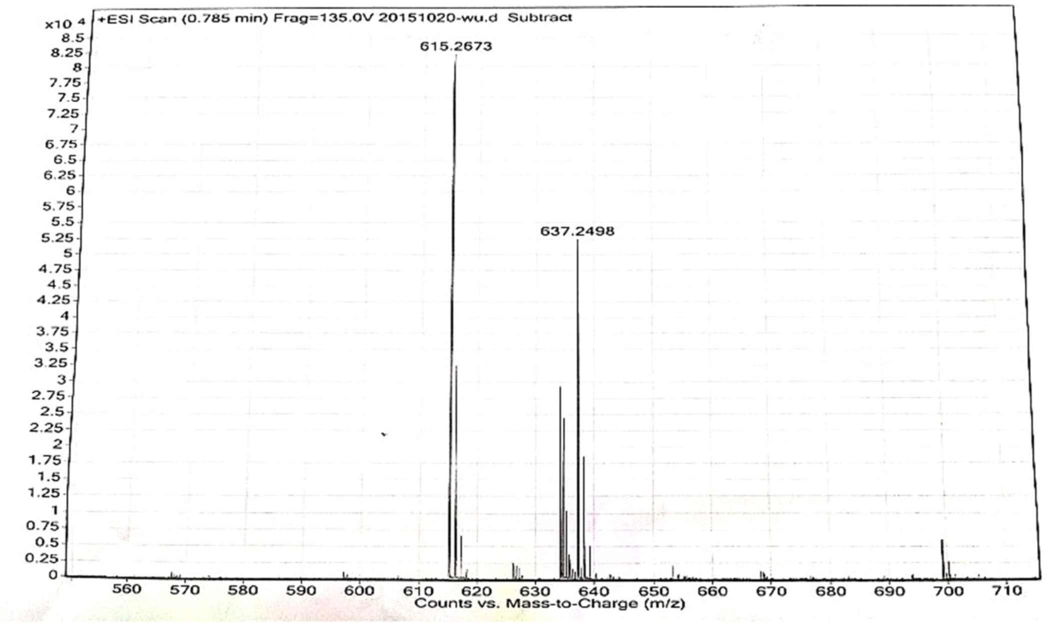

Figure S19 High resolution mass spectrum of probe **CPT**.

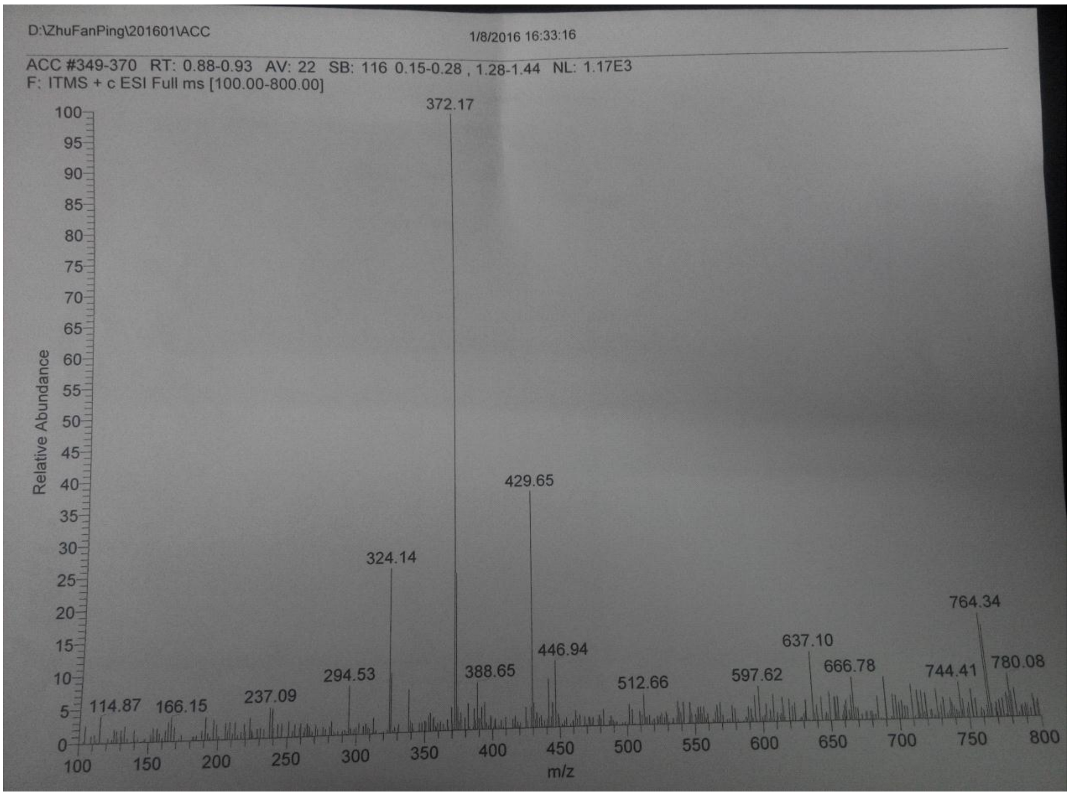

Figure S20 Mass spectrum of the acceptor.

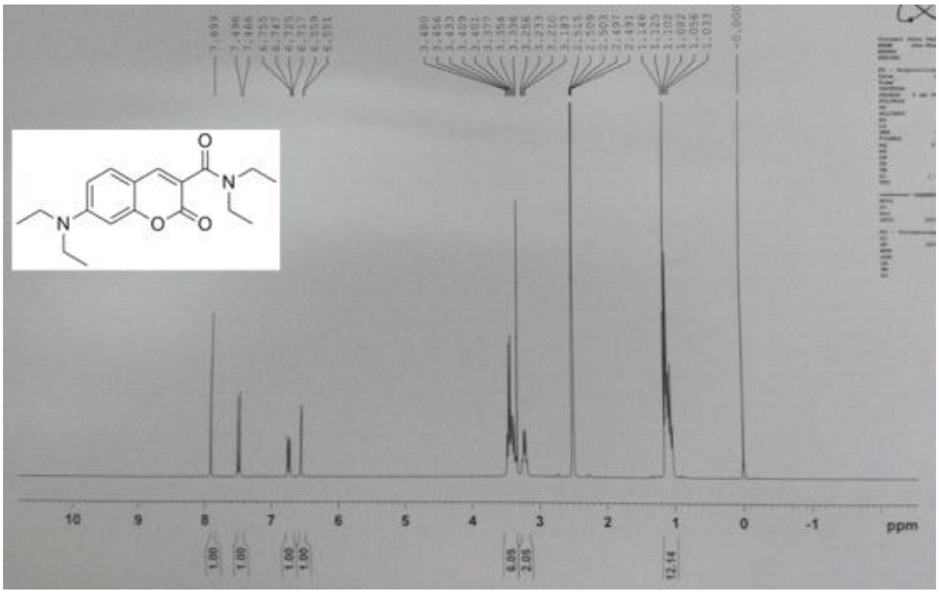

Figure S21 ^{1}H NMR spectrum of the donor (DMSO-d_6).

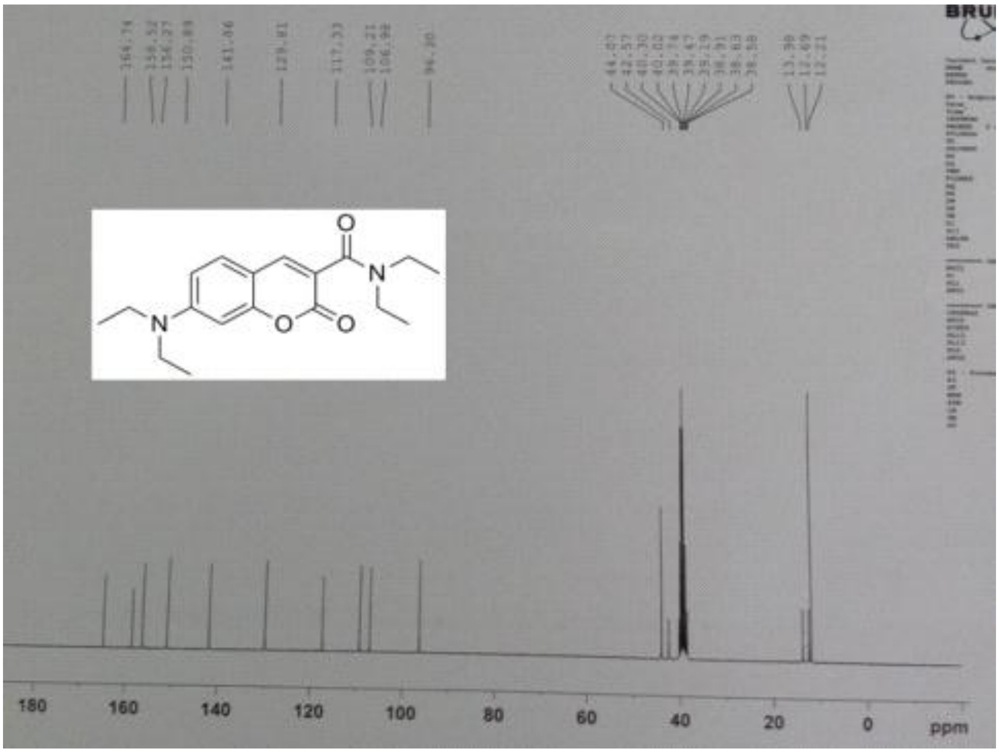

Figure S22 ^{13}C NMR spectrum of the **donor**.

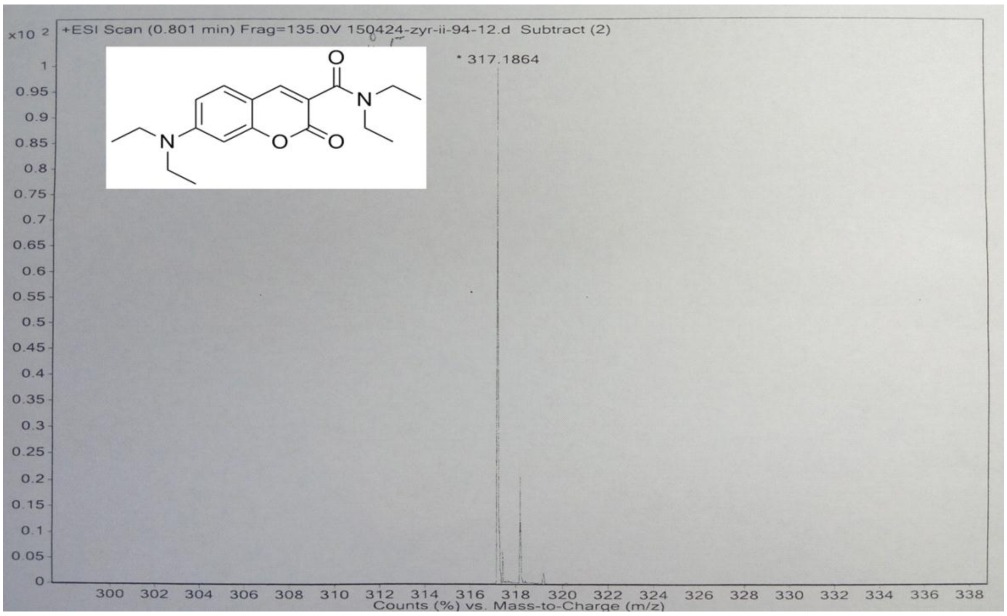

Figure S23 High resolution mass spectrum of the donor.

Table S1. Comparison of ratiometric fluorescent probes for HSO_3^-/SO_3^{2-}.

Probe structures	λ_{ex} (nm)	λ_{em} (nm)	Limit (nM)	Response time	Equiv. of HSO_3^-	Ref.
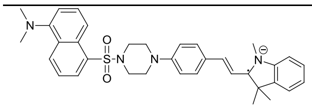	410	530/ 580	100	120 s	6.0	[3]
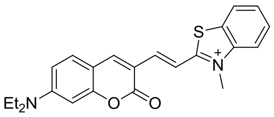	445	475/ 635	380	5 min	10	[4]
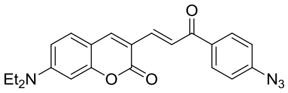	410	460/ 590	100	1 h	10	[5]
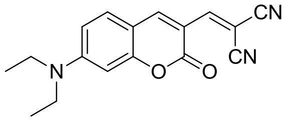	449	480/ 578	580	30 s	200	[6]
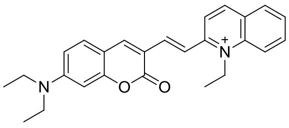	450	518/ 610	89	15 min	200	[7]
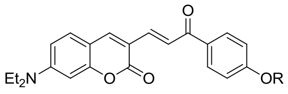	410	465/ 592	200	$t_{1/2} \approx$ 5 min	50	[8]

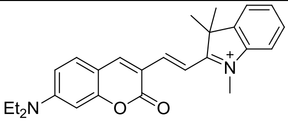	405	480/ 650	90	30 min	100	[9]
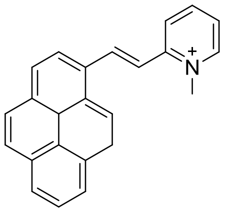	340	376/ 395	2760	20 min	500	[10]
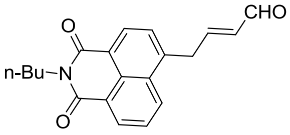	439	535	100	5 min	30	[11]
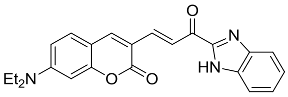	415	395/ 492	53	50 min	10 (CTAB 1 Mm)	[12]
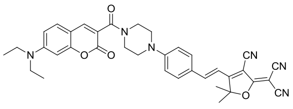	370	470/ 632	45	60 min	10	this work

References

1 Wu, M. Y., Li, K., Li, C. Y., Hou, J. T. & Yu, X. Q. A water-soluble near-infrared probe for colorimetric and ratiometric sensing of SO_2 derivatives in living cells. *Chem. Commun.* **50**, 183-185, (2014).

2 Zhang, Y. R., Meng, N., Miao, J. Y. & Zhao, B. X. A Ratiometric Fluorescent Probe Based on a Through-Bond Energy Transfer (TBET) System for Imaging HOCl in Living Cells. *Chem. Eur.J.* **21**, 19058-19063, (2015).

3 Li, D. P., Wang, Z.Y., Cao, X, J., Wang, X., Cui, H. Z., Miao, ,J. Y. & Zhao, B. X.,. A mitochondria-targeted fluorescent probe for ratiometric detection of endogenous sulfur dioxide derivatives in cancer cells. *Chem. Commun.*, DOI: 10.1039/C1035CC09092J.

4 Sun, Y. Q., Liu, J., Zhang, J., Yang, T. & Guo, W. Fluorescent probe for biological gas SO2 derivatives bisulfite and sulfite. *Chem. Commun.* **49**, 2637-2639, (2013).

5 Tian, H. *et al.* A coumarin-based fluorescent probe for differential identification of sulfide and sulfite in CTAB micelle solution. *Analyst* **139**, 3373-3377, (2014).

6 Wu, M. Y. *et al.* A real-time colorimetric and ratiometric fluorescent probe for sulfite. *Analyst* **138**, 3018-3025,

(2013).

7 Tan, L., Lin, W., Zhu, S., Yuan, L. & Zheng, K. A coumarin-quinolinium-based fluorescent probe for ratiometric sensing of sulfite in living cells. *Org. Biomol. Chem.* **12**, 4637-4643, (2014).

8 Tian, H., Qian, J., Sun, Q., Bai, H. & Zhang, W. Colorimetric and ratiometric fluorescent detection of sulfite in water via cationic surfactant-promoted addition of sulfite to alpha,beta-unsaturated ketone. *Anal. Chim. Acta.* **788**, 165-170, (2013).

9 Xu, W. *et al.* A mitochondria-targeted ratiometric fluorescent probe to monitor endogenously generated sulfur dioxide derivatives in living cells. *Biomaterials* **56**, 1-9, (2015).

10 Xu, G., Wu, H., Liu, X., Feng, R. & Liu, Z. A simple pyrene-pyridinium-based fluorescent probe for colorimetric and ratiometric sensing of sulfite. *Dyes Pigments* **120**, 322-327, (2015).

11 Sun, Y. Q., Wang, P., Liu, J., Zhang, J. & Guo, W. A fluorescent turn-on probe for bisulfite based on hydrogen bond-inhibited C=N isomerization mechanism. *Analyst* **137**, 3430-3433, (2012).

12 Dai, X. *et al.* An effective colorimetric and ratiometric fluorescent probe for bisulfite in aqueous solution. *Anal. Chim. Acta.* **888**, 138-145, (2015).
